# Chemical Mapping for Insight into Early 1900s Historical Photographic Films

**DOI:** 10.1021/acsami.5c02952

**Published:** 2025-03-25

**Authors:** Alessandro Auditore, Nunzio Tuccitto, Valentina Spampinato, Paola Benedetta Castellino, Alberto Torrisi, Antonino Licciardello

**Affiliations:** † Department of Chemical Sciences, Università degli Studi di Catania, viale A Doria n 6, Catania 95125, Italy; ‡ CSGI - Center for Colloid and Surface Science, viale A Doria n 6, Catania 95125, Italy

**Keywords:** hyperspectral imaging, principal component analysis (PCA), ToF-SIMS (time-of-flight secondary ion mass spectrometry), gelatin degradation, photographic film preservation

## Abstract

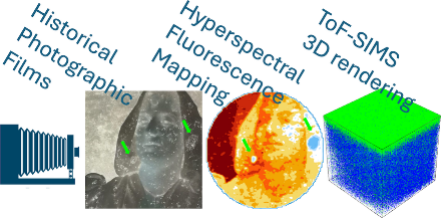

This study examines the degradation mechanisms of early 1900s gelatin-based photographic films, integrating advanced analytical techniques to provide a comprehensive understanding of the structural and chemical changes within these materials. Ultraviolet hyperspectral fluorescence-induced visible fluorescence mapping (HUVFM) revealed fluorescence quenching in degraded regions, indicative of protein and collagen degradation in the gelatin matrix. Principal component analysis (PCA) revealed distinct spectral responses in these areas, supporting the hypothesis of gelatin degradation. Time-of-flight secondary ion mass spectrometry (ToF-SIMS) clarified the molecular and elemental composition, confirming the removal of the protective paraffin lacquer in the degraded areas and exposing the underlying gelatin. Depth profiling further demonstrated the spatial distribution of degradation products. In addition, the study examined opaque areas related to silver mirroring, proposing the oxidation-migration-reaggregation model as a plausible explanation for this phenomenon. The results highlight the interaction between chemical processes and structural deterioration in gelatin-based photographic films, offering essential insights into conservation strategies.

## Introduction

Gelatin emulsion photography became popular in the 19th century.^1^ The gelatin dry plate process was introduced by Richard Leach Maddox in 1871 eliminating the need to prepare and develop plates while still wet like in the case of wet collodion process. These included greater sensitivity, longer shelf life, and improved convenience compared to wet collodion. Gelatin emulsion-based photography contributed to a more standardized and reproducible photographic processes, enabling the development of various photographic formats, including glass negatives and roll film. Dry plates made photography more flexible and convenient, allowing photographers to carry prepared plates and extend the range of subjects they could capture. The subsequent introduction of flexible film made with gelatin emulsion by George Eastman and his company Kodak in the late 1880s further revolutionized photography. This marked the transition from glass plates to portable, flexible film, making photography definitively accessible to a wider public before the digital revolution.

Gelatin-based photographic films from the early 1900s have become highly sought after by collectors and institutions, due to their historical significance and scarcity. Auctions and exhibitions often feature these artifacts, further emphasizing their value. Certainly, both old-type photographs face specific challenges related to degradation over time. Gelatin emulsions can suffer from cracking and peeling over time, leading to the loss of image layers. This is often exacerbated by fluctuations in temperature and humidity. Silver mirroring, where metallic silver particles migrate to the surface of the emulsion, can occur. This phenomenon manifests as a reflective or iridescent appearance on the photograph’s surface. Preserving these valuable artifacts requires a delicate balance between making them accessible for study and ensuring their long-term survival. Several research groups studied photo degradation, including that of photographs from the early 1900s. The intricate process of silver mirroring formation in black and white (B&W) photographs was investigated by Di Pietro.^2^ The foundation of B&W photographs lies in a gelatin emulsion containing silver halide crystals, which transform into silver grains during processing. These grains are typically found on support materials such as paper or glass. They have micrometric dimensions and exhibit complex dendritic structures, which make them susceptible to reactions and corrosion. Typically, there are three different types of degradation in black and white photos observed: a shift toward a yellow tone, the emergence of red spots, and the development of silver mirroring, a bluish metallic sheen, primarily found at photograph edges. The exploration of silver mirroring dates back to 1882, attributing its occurrence to hydrogen sulfide. The Oxidation Migration-Reaggregation model (OMR), proposed in 1963, assumes that silver ions undergo migration within the gelatin, reaggregating either as red spots or forming silver mirroring at the surface. Experiments were conducted on silver gelatin glass negatives from the Cueni study collection (1910–1920s).

Spectroscopic analyses, employing techniques such as X-ray Diffractometry (XRD) and X-ray Photoelectron Spectroscopy (XPS), were employed to discern the chemical composition of silver mirroring. XRD analysis confirmed silver mirroring’s composition as silver sulfide (Ag_2_S), while XPS analysis revealed the presence of carbon, oxygen, silver, sulfur, iodine, and mercury in the mirrored areas.^3^ Notably, particles beneath the main layer exhibited a predominantly spherical shape. Thus, the chemical composition of silver mirroring particles was affirmed to be silver sulfide. Conservators and institutions play a crucial role in the ongoing effort to safeguard the rich history encapsulated in these early photographic treasures. Exploring cleaning methods in the restoration of 19th−20th century photographs is an innovative and uncharted area. Romani et al. reported about two distinct cleaning approaches to address degradation issues.^4^ Specifically, calcium chloride was applied to eliminate the silver mirroring effect on a gelatin silver print. To preserve 19th and twentieth-century photographs, the application of various characterization techniques plays a fundamental role.^5^ Conservation scientists have explored various treatment methods, including atmospheric pressure plasma, to stabilize and potentially remove degradation without altering the underlying image. High spatial resolution characterization techniques like TEM have been employed to assess the efficacy of these treatments and understand the degradation process.^6^ The use of such techniques allows direct observation of changes in the corrosion layer after treatment, without influencing the characterization of individual nanoparticles or altering the spatial distribution of corrosion in the soft matrix and on the surface of the negative. Sessa et al. determined the amount of sulfur by X-ray fluorescence spectroscopy, to understand whether the presence of this element was due to the artistic technique used or to a deterioration process.^7^ Basso et al. focused on photographs from 1894 to 1897 underwent analysis using a combination of noninvasive and microinvasive techniques, such XRF, fiber optics reflectance spectroscopy, Raman, and Fourier-transform infrared spectroscopies, and scanning electron microscopy with energy-dispersive X-ray spectroscopy.^8^ Similarly, data collected by Zamboni et al. provides curators and historians with fundamental information for cataloging.^9^ The degradation of gelatin-based photographic films is a subject of significant importance not only for understanding the inherent chemical and physical processes affecting organic materials but also for the practical implications in the conservation of cultural heritage. Several studies^10−15^ have demonstrated that the deterioration of these films can lead to the loss of unique historical information. Thus, understanding the degradation mechanisms at a molecular level is essential for developing targeted conservation strategies. Moreover, the interdisciplinary approach combining analytical chemistry and conservation science offers new insights into the preservation of valuable archival materials. A significant limitation of the findings up so far is the dichotomy between methodologies: very high-resolution techniques focus on the minutiae of the artifacts, while low-resolution approaches are employed to analyze the artifact as a whole. This division often results in a fragmented understanding of the degradation mechanisms, as it fails to bridge the gap between detailed chemical insights and the broader spatial context of the artifact. To address this, we propose a combined multiscale approach, integrating Hyperspectral Ultraviolet Induced Visible Fluorescence Mapping (HUVFM) and Time-of-Flight Secondary Ion Mass Spectrometry (ToF-SIMS), to provide a comprehensive analysis that seamlessly connects microscale chemical processes with macroscale spatial distribution.

Ultraviolet Induced Fluorescence Imaging has emerged as a valuable tool for scientifically examining and conserving cultural heritage objects, offering a noninvasive approach to delve into material composition and condition.^16−19^ In contrast to conventional fluorescence spectroscopy, which typically provides analytical information averaged over a relatively large portion of the sample, space resolved fluorescence spectroscopy concentrates on gathering data about emitted light from distinct spatial points on the sample’s surface. This methodology facilitates the creation of spatial maps of luminescence, unveiling variations in light emission throughout the sample. We incorporated a three-degree-of-freedom automatic manipulator for optimal positioning of the optical probe. Each point in the hyperspectral image corresponds to a spectrum, furnishing intricate details about the sample’s light response at diverse wavelengths when exposed to UV light. To achieve a more refined characterization of degradation at the microns scale, we utilized the ToF-SIMS technique. The ToF-SIMS analysis, guided by the HUVFM results, allowed for a detailed examination of the chemical composition and structural changes in the degraded samples. ToF-SIMS has proven to be a powerful tool in the field of cultural heritage, providing valuable chemical and structural information that contributes to the understanding, preservation, and interpretation of diverse cultural artifacts.^20−24^ ToF-SIMS has been employed, late 1990s and early 2000, for the characterization of silver halide microcrystals in contemporary photography materials^25^ but the application to ancient early 1900s photography appears to be limited based on the authors’ knowledge. Previous studies^26,27^ have primarily focused on the macroscopic effects of environmental factors on gelatin-based films, with limited emphasis on the molecular degradation mechanisms. The current study employs ToF-SIMS and complementary techniques to offer a detailed molecular perspective, thereby filling a critical gap in the literature. ToF-SIMS has evolved over the years, and recent advancements include the introduction of ion beams based on clusters of atoms. This innovation enhances the performance of ToF-SIMS, offering improved sensitivity and resolution in materials analysis. The ability to use cluster ion beams provides a more gentle and efficient means of sputtering of organic target, which is particularly beneficial when dealing with delicate and aged samples, such as those found in ancient early 1900s photography. In this study, we investigate the degradation of gelatin-based photographic films using advanced spectrometric techniques. The methodological framework and sample details, including historical background, are described in detail in the Materials and Methods section

## Materials and Methods

The analyzed samples, originating from archival collections dating back to the early 20th century, provide a unique opportunity to study the long-term degradation processes of gelatin-based films. Their historical context is integral to understanding the conservation challenges faced by such materials. The photographic films utilized in this study originate from two distinct collections. One set comes from a private collection, generously donated to the research entity. Another sample, of notably higher value, consists of a fragment sourced from the Photographic Archive of the Superintendence for Cultural and Environmental Heritage of Syracuse (Italy). The photographic corpus, which comprises positives, negatives, slides, digital files, and a small film library, represents a collection of immense historical, artistic, and documentary significance. A particularly valuable part of this corpus is the Rosario Carta Photographic Fund. Discovered in 2011 within a drawer of an old wooden cupboard in the Fontana corridor, the find was immediately recognized for its unprecedented historical relevance. However, it was also apparent that the haphazard placement of these negatives, spanning half a century, had jeopardized their accessibility, proper preservation, and use, crucial steps in establishing a modern archive aimed at grounding the historical, cultural, and anthropological identity of a civilization. The chronology of the Photographic Fund spans from the early 1900s to 1950. Our study benefited significantly from the structured information on the parchment paper envelopes containing the negatives. These envelopes provided details on the subject, the date on which the picture was taken, and the photographer’s name, as documented in the Carta inventory of the negatives. This information unequivocally attributed the Photographic Fund to Rosario Carta himself. The (accidental or deliberate) isolation of this group of negatives excluded it from the comprehensive reordering of all the negatives in the Photographic Archive conducted in 1952. The Rosario Carta Photographic Fund comprises 534 films (522 with glass backing and 12 with cellulose nitrate backing) in 180 × 240, 130 × 180, and 100 × 150 mm^2^ formats. These documented various sites, archeological excavations in Sicily, archeological finds, and aspects of daily life. Taken on glass plates or cellulose nitrate during Carta’s work trips, these photographs collectively serve as a rich source of diverse and significant information, capturing moments in the history of archeology and beyond. The methodology we propose here aims to contribute to the broader scientific understanding of the degradation processes affecting photographs from the early 1900s. By employing advanced analytical tools, we seek to unravel the complex interplay of factors leading to the deterioration of these cultural heritage artifacts. Such insights are crucial for developing effective conservation strategies that can mitigate further degradation and ensure the long-term preservation of these valuable historical items.

**Figure 1:**
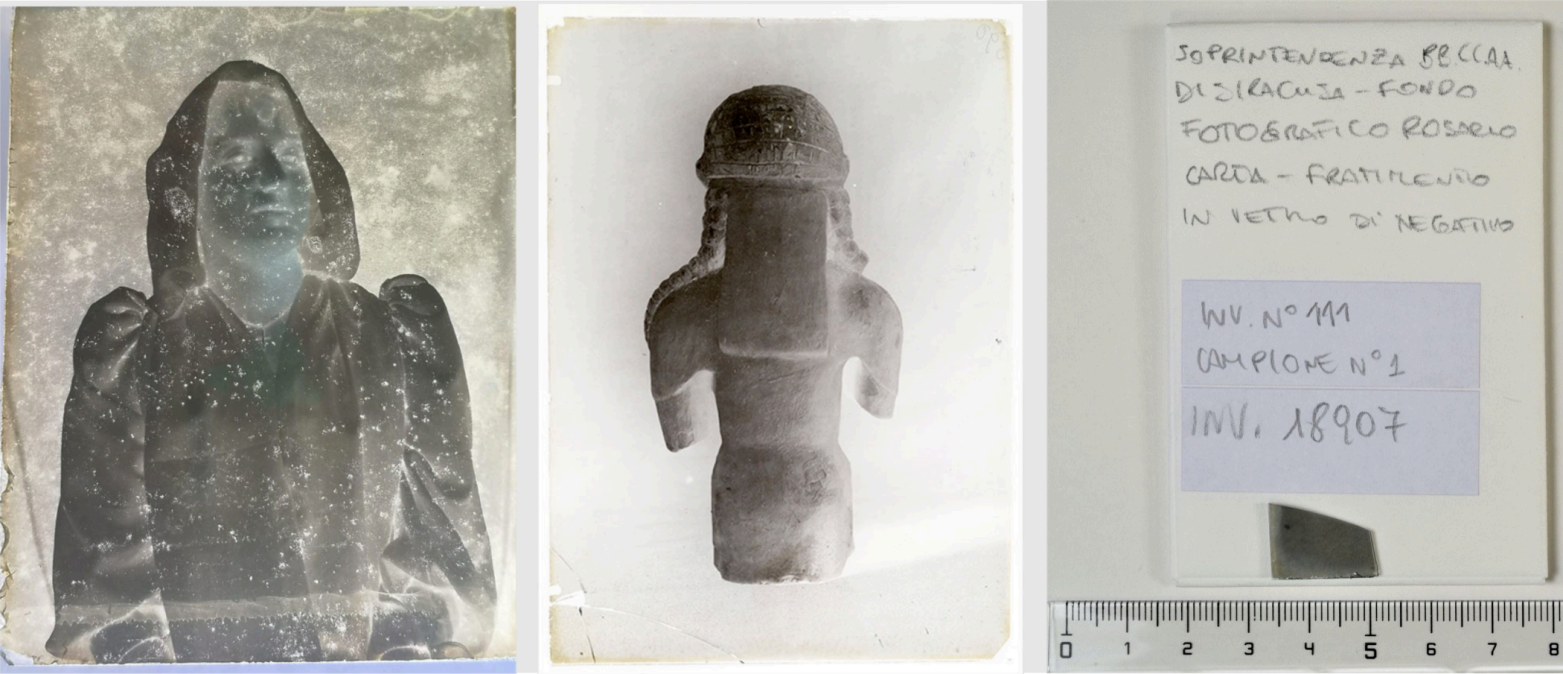
Optical photographs of the samples under investigation. Sample 1 (left) depicting a young woman from the early 1900s, a find known by the name “A signurina” (”the young lady”). Specimen 2 (middle) depicting “Pupidda David” (“David Puppet”). Detail of specimen 2 (right), the fragment under analysis.

Figure 1 displays the two photographic films employed in this investigation. Figure 1a presents Sample1, the photographic film named as “A signurina” (aka “The young lady”) sourced from a private collection generously donated to the research entity. Figure 1b features Sample2, obtained from the Photographic Archive of the Superintendence for Cultural and Environmental Heritage of Syracuse (Italy). Figure 1c provides a close-up view of the analyzed fragment from the bottom left portion, noting that the fragment was already broken. The so-called “Pupidda David” (aka “David Puppet”) was bought by archeologist Paolo Orsi for the National Archaeological Museum in Syracuse (Italy) taking its name from the family from which he purchased it.

The characterization of HUVFM was conducted using a custom-built instrument. The analysis probe consists of a bundle of 19 Y-shaped fibers (BF19Y2HS02 sourced from Thorlabs), positioned 1 mm away from the analysis point. Among the 19 fibers, 10 are Y-ends connected to the source, which is a 365 nm LED (Light Emitting Diode) sourced from Thorlabs. The remaining 9 fibers are linked to an optical block housing bandpass filter sourced from Thorlabs, designed to block backscattered light from the source. Subsequently, the CCD detector (CCS100/M sourced from Thorlabs) is connected to a bundle of optical fibers arranged linearly (BFL200HS02 sourced from Thorlabs), maximizing the light collected by the sampling slit of the detector. The fiber movement over the sample is facilitated by a three-axis micromanipulator, driven by a stepper motor (Ender 3 sourced from Creality), and programmatically controlled by a microcontroller (ATMmega328p based). We have made the motion and acquisition software, developed in Python, available. Principal Component Analysis (PCA) of the spectra was conducted using Python NUMPY and SCIPY libraries. The conversion of the spectrum to RGB color was executed through the Python COLOUR library, utilizing CCS-ILLUMINANTS[’cie-2–1931’][’E’]. Images and plots were generated using the Python MATPLOTLIB library. The scripts for these processes were developed by us. The SIMS experiments were carried out using a ToF-SIMS instrument (ToF-SIMS IV, ION-TOF GmbH, Münster, Germany). As analysis gun, 25 keV liquid metal ion gun (LMIG) operating with bismuth primary ions was used. The surface spectra were acquired in positive polarity mode using Bi_3_^+^ primary ions from square areas of 500 × 500 μm^2^ in high current bunched mode (resolution M/ΔM *>* 7000 at *m*/*z* = 28), with a beam spot size of ∼ 2.5 *μm*. Large area images were acquired in the so-called stage-raster mode. Depth profiles were acquired by a dual beam configuration, using Ar_500_^+^ at 20 keV as sputter beam, raster scanned over 500 × 500 *μm*^2^ area and the same analysis beam used for the surface spectra acquisition, raster scanned over a 100 × 100 *μm*^2^ concentric to the sputter beam scan area. During the analysis, charging of the surface was prevented by applying charge compensation using low-energy (∼ 20 eV) electron flood gun. Spectral interpretation was carried out using SurfaceLab software v6.7 (ION-TOF GmbH, Münster, Germany). Profilometer analysis was performed by P-7 Stylus Profiler KLA-Tencor with 315 *μm* of scan depth.

## Results and Discussion

**Figure 2:**
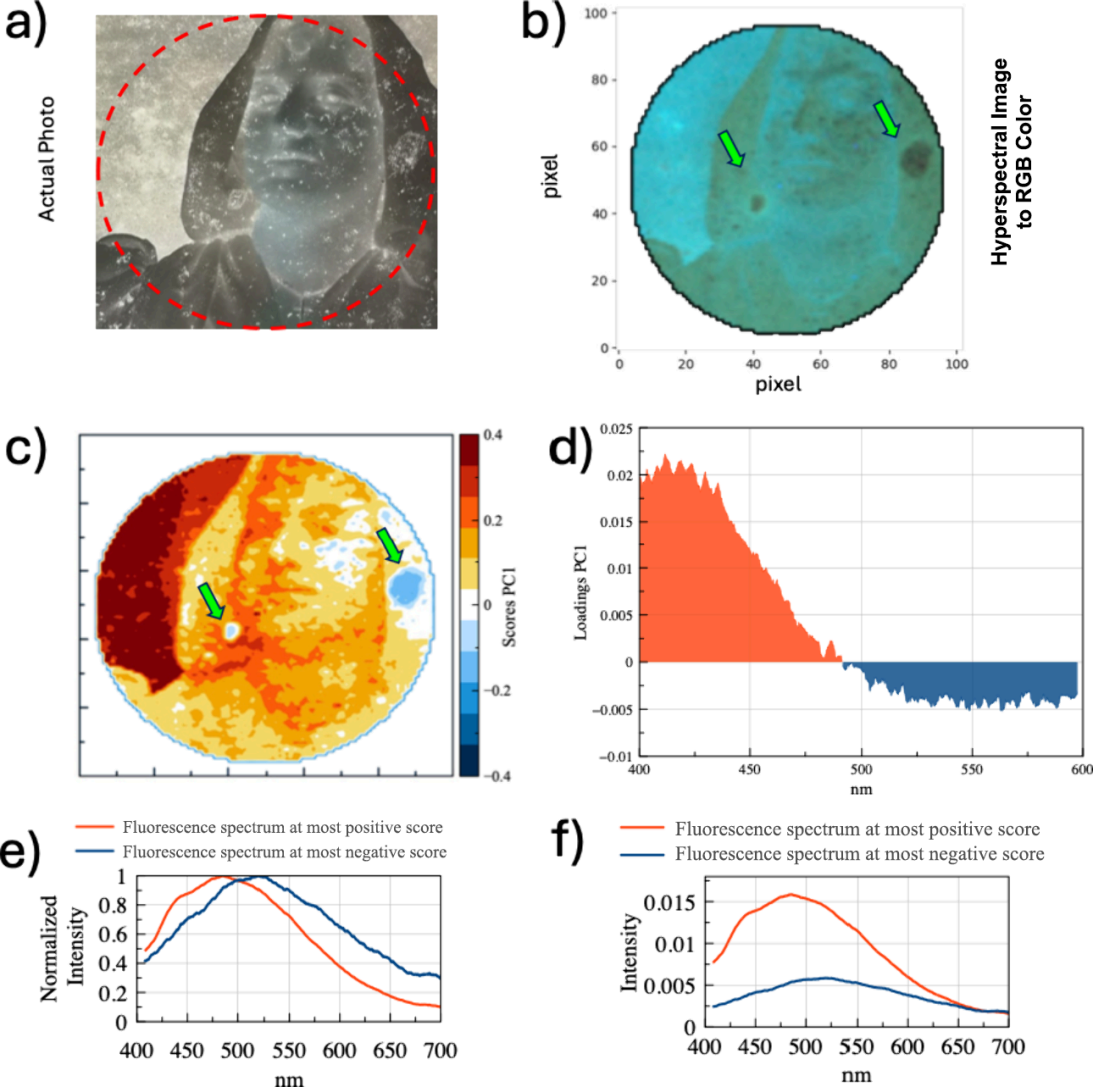
Sample 1. Results of the HUVFM investigation. (a) Actual optical picture of the area under investigation. The red circle outlines the region of interest. (b) Reconstructed image derived from transformation of a hyperspectral image into an RGB color image. (c) False-color image derived from the PC1-SCORES values obtained through PCA applied to the hyperspectral image after normalization to the total integrated fluorescence intensity. (d) Normalized fluorescence spectra acquired from pixels having the most positive PC1 scores (red) and the most negative PC1 scores (blue). (e) Actual fluorescence spectra acquired from pixels having the most positive PC1 scores (red) and the most negative PC1 scores (blue). (f) Normalized fluorescence spectra acquired from pixels having the most positive PC1 scores (red) and the most negative PC1 scores (blue).

In this study, two distinct types of gelatin-based photographic films were analyzed. Although the chemical composition and degradation pathways differ between the samples—necessitating the use of tailored analytical methods—the overall objective is to elucidate the key degradation mechanisms and identify any common features. The first sample, characterized by clear signs of chemical and physical degradation, was examined using a combination of two complementary techniques such as HUVFM and ToF-SIMS, whereas the second sample, which exhibits chemical degradation related to the so-called silver mirroring, was subjected to ToF-SIMS including 3D-depth profiling. Together, these investigations provide a comprehensive picture of the degradation phenomena, highlighting both sample-specific and universal findings relevant to the conservation of photographic materials. Figure 2 presents a summary of the findings from the HUVFM investigation on Sample 1. Figure 2a displays the actual optical image, obtained from the gelatin side, with the designated analysis area superimposed. Figure 2b illustrates the reconstructed image derived from transforming hyperspectral images captured in the darkroom under UV irradiation into an RGB Color Image. This image closely mirrors the optical image in the visible spectrum. Remarkably, the regions exhibiting notably diminished fluorescence, as indicated by the green arrows, draw attention to areas of special significance. The fluorescence observed at this specific wavelength is predominantly linked to the protein component within the gelatin. Consequently, these darkened areas stand out as clear indicators of degradation within the gelatin structure, emphasizing the need for focused attention to understand and address the underlying deterioration processes. The peculiar spectral response in these low-fluorescent portions suggests alterations or degradation in the proteinaceous matrix of the gelatin, prompting further investigation into the nature and extent of these changes for comprehensive preservation efforts. Figure 2c present false-color images derived from the SCORE values obtained through Principal Component Analysis (PCA) applied to the hyperspectral HUVFM images. Prior to performing PCA, each pixel’s fluorescence spectrum was normalized to its total integrated intensity. This normalization is crucial because it prevents the PCA from extracting features solely related to absolute fluorescence intensity, which can be readily determined by direct integration. Instead, the PCA emphasizes more subtle variations in the spectral shape that are indicative of chemical or physical degradation. The PCA results are further elucidated by examining the loadings associated with the principal components. Figure 2d shows the “pseudo-spectrum” of PC1 loadings plotted as a function of wavelength. In this representation, the loadings reflect the contribution of each wavelength to the variance captured by PC1. Notably, areas potentially identified as degraded (characterized by negative PC1 scores) are associated with negative loadings above 490 nm, while regions without apparent degradation (positive PC1 scores) exhibit positive loadings below 500 nm. This distinction provides a quantitative basis for identifying and mapping spectral shifts due to degradation. Figure 2e-f illustrate a comparison between the spectra acquired from areas with extreme PC1 scores. The spectrum corresponding to the maximum PC1 score (typical of nondegraded regions) shows a peak emission near 470 nm, whereas the spectrum from the minimum PC1 score (characteristic of degraded areas) displays a red-shifted peak near 510 nm. Additionally, when comparing the non-normalized spectra, a significant quenching of fluorescence is observed in the degraded regions—a result consistent with the images obtained from the univariate analysis of the hyperspectral data. This observed alteration in the fluorescence spectra is in good agreement with our hypothesis regarding gelatin degradation. Such a shift resonates with the expected fluorescence behavior associated with changes in the proteinaceous matrix of gelatin.^28^ It is known that when collagen, a primary constituent of gelatin, changes in its molecular environment fluctuations in fluorescence behavior are detected.^29^ The observed quenching and shift in emission from the gelatin matrix offers valuable insights into the ongoing degradation processes within the material. Consequently, the HUVFM investigation seamlessly aligns with the presumed collagen degradation.

**Figure 3:**
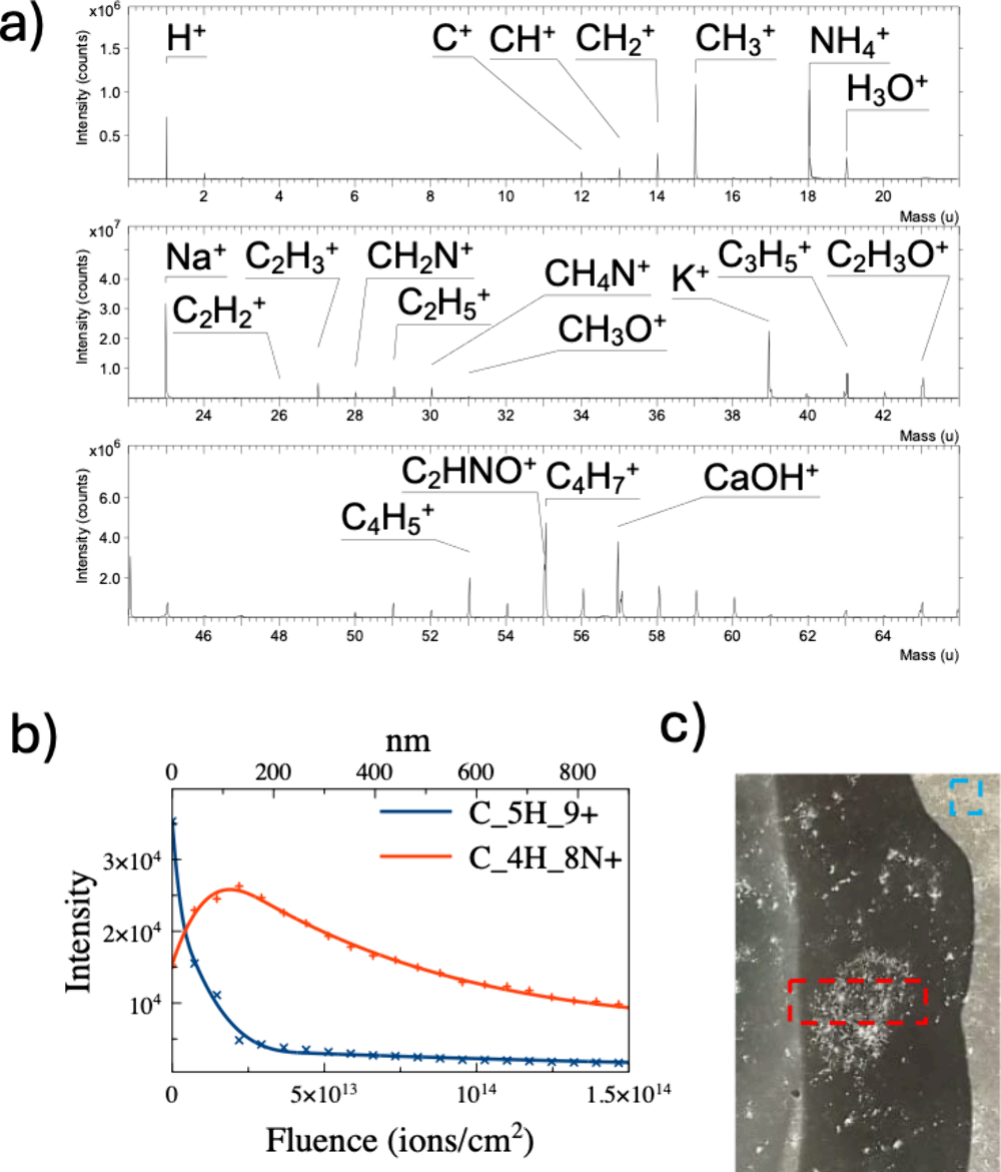
Sample 1. (a) Portion of the positive ions of the ToF-SIMS spectrum reporting the most relevant peaks. The assignation has been performed according to the ToF-SIMS reference spectra database.^31^ (b) ToF-SIMS depth profile of a region of interest free from degradation. (c) Regions of interest. The red line is the area of panel a under investigation, and the blue line is the area of panel b under investigation.

To validate this observation, we conducted additional investigations through ToF-SIMS. Figure 3a presents a segment of the ToF-SIMS spectrum, specifically showing positive ions in the region delineated by the dashed red line in Figure 3c, which corresponds to the potential degradation site pinpointed by HUVFM investigation. The assignment of the observed ion peaks was conducted by experienced operators using the reference spectra integrated within the instrument’s software. Each peak was initially matched based on its exact mass, taking into account the high mass resolution (approximately 7000 at *m*/*z* 28). To ensure accuracy, the assignments were further validated by comparing the observed isotopic distributions with those reported in the literature for similar organic compounds, such as paraffins^30^ and protein materials.^31^ This rigorous approach allowed us to confidently attribute key peaks to specific molecular fragments, thereby providing a solid basis for interpreting the chemical alterations in the sample. The assigned signals align with the expected chemical composition of the photographic film. Evident hydrocarbon fragments, indicative of the presence of the surface paraffinic protective lacquer, are visible. Additionally, peaks corresponding to protein fragments and oxidation compounds are observed.^32^Figure 3b illustrates the signals obtained from a ToF-SIMS depth profile conducted on a region free from degradation. Notably, the C_5_H_9_^+^ hydrocarbon-related peak, associated with the presence of paraffin lacquer, is pronounced at the surface but diminishes with depth indicating that was a surface protecting layers or likely due to the cumulative sputter-damage induced by the Ar_500_^+^ employed as sputter beam.

**Figure 4:**
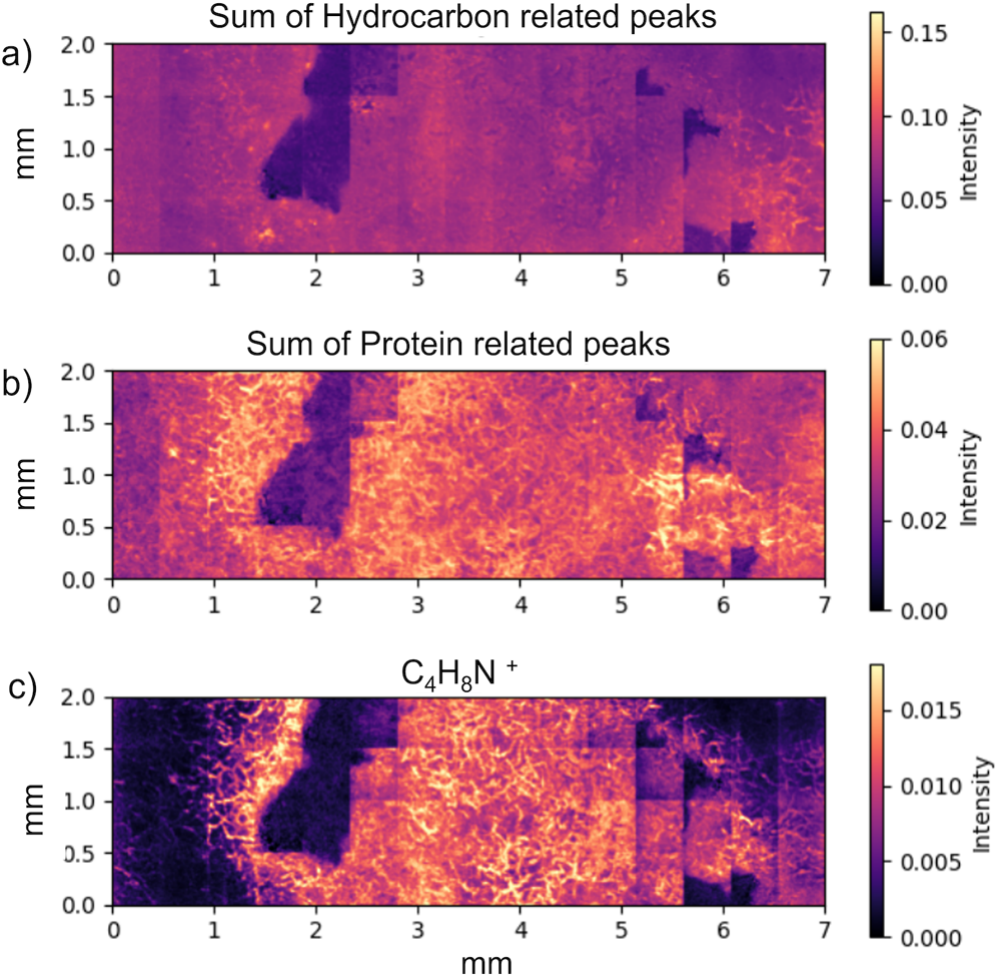
Sample 1. False-color chemical maps of positive ions acquired by ToF-SIMS using macroraster mode. Each image has been normalized by dividing by the total ion counts map in order to avoid topographic artifacts in the images. (a) Hydrocarbon-related map (sum of fragment peaks assigned to positive ions CH^+^, CH_2_^+^, C_2_H_2_^+^, C_2_H_3_^+^, C_3_H_5_^+^, C_4_H_5_^+^, C_4_H_7_^+^, and C_5_H_7_^+^). (b) Distribution map of the sum of protein-related positive ion peaks (including peaks assigned to CH_2_N^+^, CH_4_N^+^, CH_3_O^+^, C_2_H_3_O^+^, C_2_HNO^+^, C_4_H_9_N^+^, C_4_H_9_N^+^, C_3_H_4_NO^+^, and C_8_H_10_N^+^^31,33,34^) across the sample surface, highlighting the spatial variations in the protein distribution. (c) Map of C_4_H_8_N^+^ assigned to the proline fragment.

To explore the spatial distribution of these chemical components, a ToF-SIMS large area analysis was carried out. Figure 4 displays false-color chemical maps of the analyzed region. Figure 4a exhibits the current map of hydrocarbon fragments- indicating a quite uniform distribution on the surface of the sample. The sum of the protein-related ion peaks, as shown in Figure 4b, was calculated by integrating the intensities of the peaks assigned according to the database of proteinogenic amino acid reference spectra for ToF-SIMS^31,33,34^ to CH_2_N^+^, CH_4_N^+^, CH_3_O^+^, C_2_H_3_O^+^, C_2_HNO^+^, C_4_H_9_N^+^, C_3_H_4_NO^+^, C_8_H_10_N^+^ positive ions. This approach provides a comprehensive view of the overall protein degradation, allowing for a spatial comparison across different regions of the sample. Notably, the chemical maps of protein component fragments reveal different observations. The areas of lighter shading, representative of higher signal intensities, indicate a preferential localization of protein fragments within the suspected degradation region. Particularly noteworthy is the map of the C_4_H_8_N^+^ peak at *m*/*z* = 70.07 (Figure 4c). This fragment is mostly associated with proline; however, it may also originate from other amino acids such as valine or from larger peptide fragments undergoing degradation. Previous studies^35^ have reported similar assignments, highlighting the complexity of the fragmentation process. Therefore, while proline is a primary candidate, we recognize that the signal may result from a combination of sources. However, the findings prompt us to propose the following hypothesis: within the suspected degradation area, there has been a removal of the protective paraffin lacquer, exposing the underlying gelatin zone.

The detection of specific hydrocarbon fragments (sum of fragments peaks assigned to positive ions CH^+^, CH_2_^+^, C_2_H_2_^+^, C_2_H_3_^+^, C_3_H_5_^+^, C_4_H_5_^+^, C_4_H_7_^+^, C_5_H_7_^+^) indicates a relatively uniform distribution of degradation products across the sample surface. In contrast, the C_4_H_8_N^+^ protein peak exhibits a peak intensity trend, indicating that the gelatin portion, as anticipated, lies beneath the lacquer. Similar trend is recognized for other protein related peaks.

**Figure 5:**
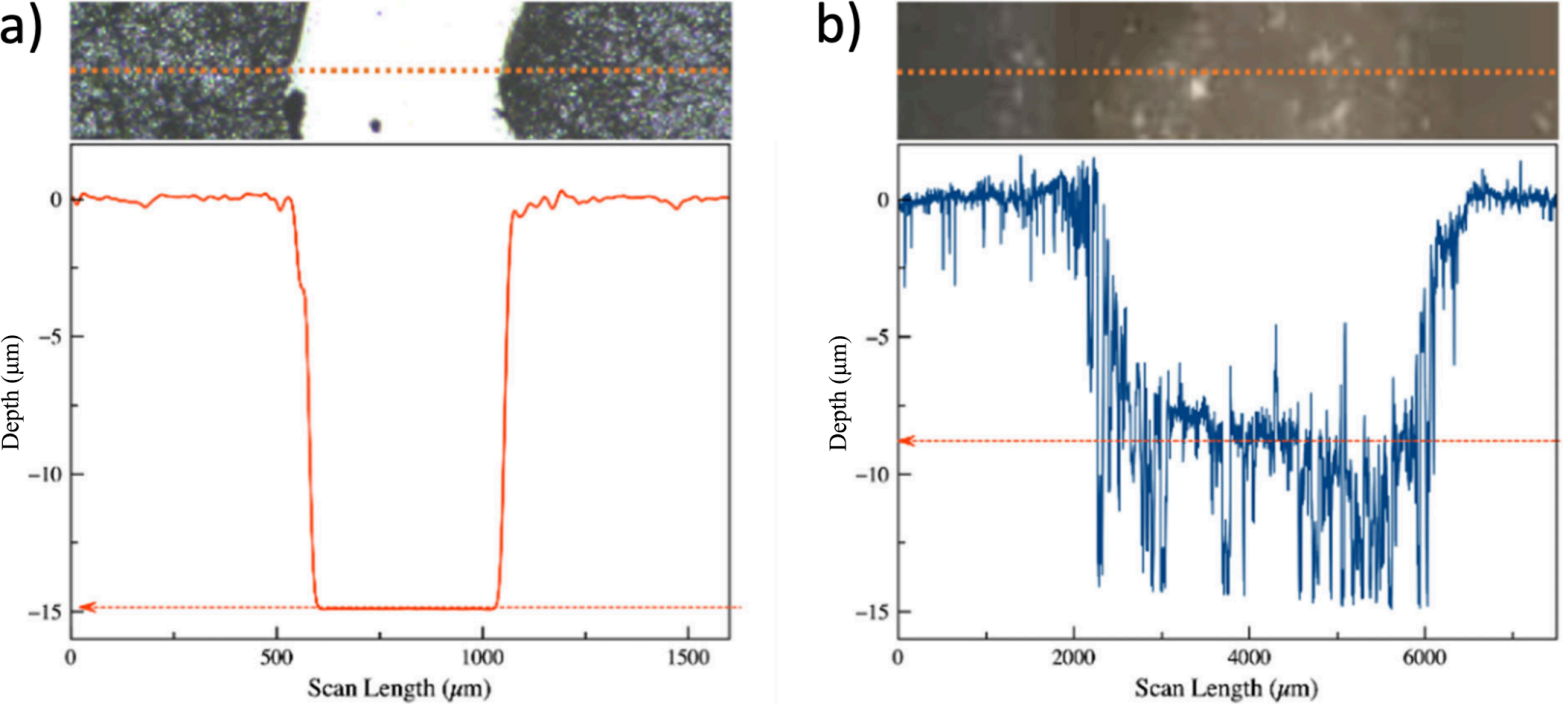
Sample 1. Profilometer step analysis of the region of interest: (a) scratch (scan step resolution of 4 μm) and (b) degradation (scan step resolution of 0.1 μm).

Figure 5 reports the height trace derived from profilometric investigation. The image in Figure 5a shows a deliberate scratch made on the gelatin with a sharp blade. Notably, the glass substrate remains unscathed by the blade. The depth analysis reveals that the film measures 15 *μm* in thickness. Conversely, the image on Figure 5b shows the trace on the area with suspected degradation. It distinctly appears as a dip, and the depth analysis indicates an approximate average depth of 8.5*μm*. This implies that approximately 50% of the film has been subjected to erosion. On closer inspection, certain areas of the film are completely eroded down to the glass substrate at a depth of 15 *μm*, as shown in Figure 5b. It is clear that the suspected degraded zone is at a lower height than the adjacent areas. This supports the conclusion that the degradation has occurred within the lower fluorescence zone, due to the lack of the protective lacquer. This removal has exposed the protein portion, which has undergone a decomposition process over time.

**Figure 6:**
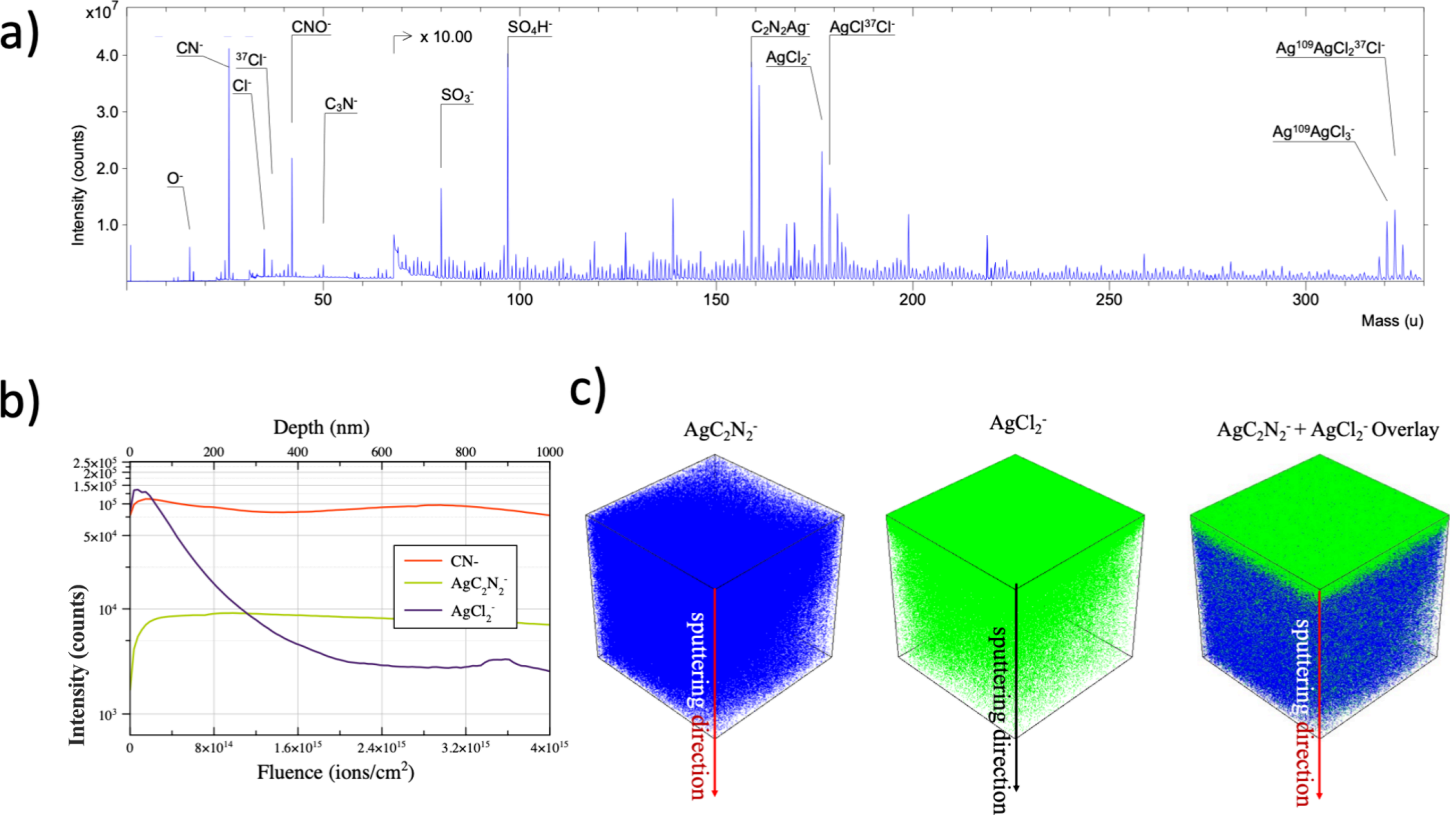
Sample 2. (a) ToF-SIMS spectrum portion of the negative ions. The most relevant peaks show the assignments upon detection of the presence of the silver mirror. (b) ToF-SIMS depth profiling showing the trends of three relevant peaks as shown in the legend. The *x*-axis shows the expressed fluence. The higher the fluence, the deeper the sampling. (c) Three-dimensional reconstruction in false colors of the AgC_2_N_2_^–^ peak intensity (blue) and AgCl_2_^–^ intensity (green). The color intensity of each voxel is related to the intensity of the peak at that point. The overlay is also shown, highlighting a non-uniform distribution of the two types of signals.

Visual investigation of Sample 2 reveals the appearance of incipient opaque zones at its edges, prompting further investigation into their nature. HUVFM characterization (not reported here) indicated that these zones exhibit fluorescence quenching. ToF-SIMS investigation was conducted, as illustrated in Figure 6, in order to provide comprehensive insights into the sample’s chemical composition. Figure 6a reports the spectrum of negative secondary ions obtained through ToF-SIMS analysis. Peak assignments, determined based on the *m*/*z* values and corroborated by isotopic abundance distribution, highlight the enrichment of silver at the surface. While these signals may indicate degradation by silver mirror formation, it is difficult to distinguish them from peaks associated with silver halide microcrystals inherent in the photographic process. To address this, a thorough analysis of the gelatin was undertaken. The silver mirror formation process involves a redox reaction triggered by the infiltration of atmospheric gases and moisture into the gelatin, primarily manifesting in the outer zone of the photographic film. Conversely, the distribution of silver microcrystals appears more evenly distributed within the gelatin, albeit with variations in size according to the amount of irradiation received during the photoexposure phase. It is important to note that the overall process is influenced by the specific composition of the photographic emulsion, the presence of other chemicals, and external environmental factors. In addition, the migration of silver ions is a key step, and the driving forces for this migration can vary depending on the conditions of the photographic material and its exposure to external elements. The mechanism known as oxidation–migration–reaggregation model is widely accepted as the underlying process.^36^ In this model, the initial step involves the oxidation of image silver particles, leading to the formation of silver ions. Subsequently, these silver ions migrate toward the top surface of the emulsion, giving rise to silver mirroring. This phenomenon has been substantiated by TEM, XPS and XRD observations revealing that surface particles were exclusively present in mirrored regions, with smaller particles situated beneath the top layer of closely packed mirroring particles.^3^ Notably, the concentration and size of these smaller particles diminish as the distance from the surface increases. This particle distribution pattern serves as compelling evidence for the migration of silver salts toward the surface. It is important to note that while this distribution is well-established, the driving force for such salt migration has not yet been identified. This collective body of evidence reinforces the validity of the oxidation–migration–reaggregation model as the predominant mechanism governing the production of silver mirroring in photographic emulsions. Figure 6b illustrates the depth profile which offers a detailed understanding of the spatial distribution of chemical constituents within the sample, emphasizing the specific localization of silver-containing areas. In fact, the intensity pattern of the Ag-related peaks reveals a distinct spatial distribution, indicating that peaks associated with the silver mirroring, like AgCl^–^, are predominantly concentrated on the outer surface of the sample. Notably, the thickness obtained from the depth profile of the silver mirror is on the order of 100–200 nm. This value is compatible with the visible light iridescence phenomena that are typically associated with this type of degradation. In contrast, gelatin-related peaks, like CN^–^ or AgC_2_N_2_^–^, exhibit a more uniform distribution throughout the depth of the sample. Figure 6c reports the 3D-reconstruction of the AgCl_2_^–^ and AgC_2_N_2_^–^ ion signals, which intensity is represented in a false color scale. Voxels are color-coded, with light blue or green indicating high intensity, dark colored voxels signifying areas with low peak presence, and transparent voxels indicating peak intensity less than 3 counts per voxel. The 3D-reconstruction serves as a visual confirmation, providing a comprehensive depiction of the spatial distribution of the two types of silver related ion peaks. Notably, the reconstruction confirms that the peak AgC_2_N_2_^–^ is mainly present at the outer surface of the sample. The visual representation confirms that the nature of the degradation can be explained by a silver mirror formation on the surface. The overlay of AgC_2_N_2_^–^ (blue) and AgCl_2_^–^ (green) shows the presence of AgC_2_N_2_^–^- deeper across all sampled depths, providing clear evidence that the degradation process does not extend uniformly throughout the entire sample depth. This observation supports the understanding that AgC_2_N_2_^–^ ion is related to gelatin-associated elements, emphasizing the surface nature of the silver mirror degradation present in the examined sample. ToF-SIMS characterization provides valuable insights into the complex chemical composition of Sample2, particularly in areas exhibiting fluorescence quenching, and shed light into potential degradation mechanisms involving silver compounds. The use of false-color images derived from SCORES values through PCA of HUVFM data set enhances the visualization of distinct zones within the material. This aids in effectively identifying and highlighting areas of interest, such as regions with notably diminished fluorescence, providing a clear visual representation of the degradation patterns. The application of ToF-SIMS for chemical mapping offers detailed insights into the spatial distribution of chemical components within the material. The false-color chemical maps effectively illustrate the presence of specific chemical species, such as hydrocarbon fragments and protein-related fragments, contributing to a comprehensive understanding of the material’s composition. The depth profiling using ToF-SIMS provides valuable information about the vertical distribution of chemical constituents within the sample. This approach contributes to the identification of distinct layers and the confirmation of the surface nature of certain degradation processes, such as silver mirror degradation. The innovative combination of HUVFM-PCA, and ToF-SIMS opens up opportunities for further research, particularly in exploring similar analytical approaches for different types of historical materials or artifacts. The insights gained from this study can contribute to the development of targeted preservation strategies for gelatin-based photographic films and similar materials. Understanding the degradation mechanisms enables the formulation of effective conservation approaches. This multifaceted approach allows for a comprehensive investigation of the gelatin-based photographic film at various levels, providing a detailed understanding of its degradation mechanisms. Understanding the degradation mechanisms enables the formulation of effective conservation approaches.

## Conclusions

This study presents an analysis of degradation processes affecting gelatin-based photographic films using HUVFM, PCA and ToF-SIMS to provide a multiscale understanding of the underlying chemical and structural transformations. HUVFM results reveal distinct regions of decreased fluorescence, which are related to localized protein degradation, particularly in the collagen matrix. PCA further highlights these areas by extracting spectral features that emphasize the altered chemical environment. Concomitant ToF-SIMS analysis validates these findings by identifying specific molecular and elemental signatures, notably the removal of the protective paraffin lacquer and exposure of the underlying gelatin. The findings of this study are of considerable significance for the preservation of historical photographic materials. This investigation enhances our comprehension of gelatin degradation, while concurrently providing concrete insights into the conservation of cultural heritage artifacts. Subsequent research, founded upon these findings, promises to refine conservation methodologies and contribute to the long-term preservation of these invaluable materials.
